# Lithium—Occurrence and Exposure—A Review

**DOI:** 10.3390/toxics13070567

**Published:** 2025-07-04

**Authors:** Manfred Sager

**Affiliations:** Bioforschung Austria, A-1220 Vienna, Austria; m.sager@bioforschung.at

**Keywords:** occurrence, production, recovery, isotope effects, water, soil, fertilizers, feedstuffs, human nutrition

## Abstract

This review contains a compilation of data about the occurrence, mining, refining, and biological actions of lithium, without claiming completeness of knowledge. This should give a baseline for judging future pollutions of environmental and agricultural items and human nutrition and may show still existing gaps of screening. Emerging electromobility and use of computers leads to a steep increase in Li-based batteries, which are a source of hazardous waste unless recycled. Lack of recovery methods from effluents and sewage, however, will increase pollution with soluble Li-salts from increasing mining and waste in the future; therefore, biochemical effects of levels out of ambient range have been included. Many published data are hidden in multi-element tables, including the data of the author. Mobile fractions of soils and soil-to-plant transfer, as well as retainment in animal tissues, are low. A lot of data, starting from geology via soils, plants, water, and human nutrition, lead to a largely unknown average daily intake for men. With respect to nutrition of dairy cows, the contribution of Li from water was highest among all elements investigated, but only 4% of intake. Main sources for human nutrition are mineral water and table salt. Li is not labelled on mineral water bottles, nor table salt, which are the main sources. Though some data have been gathered, for human nutrition, the average daily intake is uncertain to estimate because some mineral waters are quite high in Li.

## 1. Preface

Lithium was discovered in 1817 by Johan August Arfvedson in petalite, a tectosilicate of composition LiAl_4_Si_4_O_10_ [1].

Lithium can be determined via atomic emission; the line of 670 nm is usually selected. In inductively coupled mass spectrometry, mass 7 is used against Rh as internal standard, and the use of an ultrasonic nebulizer is possible [2].

Reference materials with certified Li contents to prove the validity of analytical data are rare. The lyophilized human serum BCR304 contains 0.985 ± 0.029 mmol/L (6.84 ± 0.20 mg/L), which is close to the toxicity level. Isotope proportions have been certified in Li_2_CO_3_ IRMM-015 (95.632% ^6^Li + 4.367% ^7^Li) and IRMM-016 (7.588% ^6^Li and 92.411% ^7^Li). Recently, beneath others, Li content in SRM1515 (apple leaves) has been published as 130 ± 23 µg/kg, in SRM1547 (peach leaves) as 140 ± 20 µg/kg, in ZC73036a (green tea) as 190 ± 30 µg/kg, in BCR-670 (aquatic plant) as 1400 ± 110 µg/kg, in GBW10015 (spinach leaves) as 1500 ± 61 µg/kg, and in GBW07603 (bush twigs) as 2500 ± 270 µg/kg [3].

After enrichment on a cation exchange column, Li isotopes can be measured by multicollector inductively coupled plasma mass spectrometry (MC-ICP-MS), which has largely improved precision compared with conventional quadrupole equipment because of simultaneous reading upon various detectors [4].

As lithium has been regarded neither as a nutrient nor as an essential or toxic metal, it is not often targeted in multi-elemental analytics, but data are rather occasionally found in multi-element screening papers, in particular in multi-element tables, without appearing in the keywords. This also happened in the datasets provided by the author.

Recently, a comprehensive review has been issued using the key word “lithium” from the databases of Google Scholar, Scopus, and Web of Science to summarize articles focused primarily on lithium [5]. Lithium has gained increasing interest because of its use in battery technology. By 2030, the EU is projected to require up to 18 times more Li than in 2024 [6].

In Portugal, lithiniferous feldspars have been primarily used by the ceramic industry to reduce the melting point of ceramic pastes [6].

In 1949, Li_2_CO_3_ was found to be beneficial in manic depressive illness [7]. Therefore, it is worthwhile to collect various data before the onset of broad use and waste spreading, which will probably happen in the near future.

Data for total digestion (e.g., HF-HNO_3_-HClO_4_) should not be mixed with aqua regia data, because for soils and sediments, expectable recoveries without HF are about half, but rather variable.

The aims of this review are to give an overview about current levels of lithium in environmental and agricultural items before the onset of possible diffuse pollution of Li-battery waste and to find a baseline for estimation of the average daily intake from human nutrition.

Unusually high numbers of self-citations are due to the fact that the author as an analyst has touched many scientific disciplines, starting from mineral prospection, sedimentology, and agriculture to human nutrition. This offers the chance to sum up all the results within a broad context.

## 2. Technical Use and Cycling

### 2.1. Mining

Currently, more than 60% of global lithium production occurs from brines, and the rest is from ores [8]. Li recovery from brines is economically feasible at Mg/Li < 10, which is probably from interactions of aqueous solutions with a granitic rock at T > 200 °C [8].

In South America, Li is extracted from brines occurring within a triangle of northwest Argentina, southwest Bolivia, and northern Chile [9].

In the Atacama Salt Flat, saline groundwater contains 0.15% Li, which serves as its major source. The brine is pumped though ponds, where halite, sylvanite, and carnallite are precipitated by evaporation by sunshine. The concentrated brine is then transported to Antofagasta to produce Li_2_CO_3_ [10].

The Puna plateau region at the Lithium Triangle in South America contains thick sequences of continental evaporites and clastic deposits fluids, possibly via intermediate formation of the clay mineral hectorite (Na_0.3_(Mg,Li)[Si_4_O_10_](OH)_2_). Li and B in the Andean salars are derived from the weathering of ignimbrites by hydrothermal fluids. The total salt concentration of the brine is on average nine times higher than sea water, and 5–50 m^3^ of fresh water is needed for washing, re-dissolution, and re-precipitation of primary Li_2_CO_3_, which causes environmental concentrations in the arid area. Further on, one ton of Li_2_CO_3_ by water evaporation produces 115 tons of water. Therefore, precipitations such as Li-aluminate, Li-Mn-spinel, or Li-titanate have been proposed, and recoveries of K and B from the brines should be considered as well. Battery-like capturing systems produce less waste but need electricity [11].

The Salar de Uyuni in the highland of Bolivia contains a high Mg/Li ratio (16–22:1), which favors losses by precipitation of Li-carnallite (LiCl·MgCl_2_·6H_2_O), Therefore, Mg is removed by slow addition of NaOH and crystallization seeds to form a filterable precipitate of Mg(OH)_2_-CaSO_4_·2H_2_O mixture at pH 8.6–11, which adsorbs B, resulting in recoveries of Mg, B, and Ca as byproducts. Further removal of Ca and Mg can be achieved by precipitation with Na-oxalate, and the Ca-Mg-oxalates can be roasted for recycling. From the resulting brine, Na and K are removed by fractionated crystallization prior to Li_2_CO_3_ precipitation [12].

In conventional evaporation of Chilenian salt brines, crystallization follows the sequence halite (NaCl + CaSO_4_·2H_2_O)—poor sylvinite (NaCl + KCl)—sylvinite (KCl)—carnallite (KCl.MgCl_2_·6H_2_O)—bischofite (MgCl_2_·6H_2_O—Li-carnallite (LiCl·MgCl_2_·6H_2_O), and the residual brine is used to precipitate Li_2_CO_3_. In order to recover the water in this arid environment, membrane distillation-crystallization has been proposed, which means the extraction of water across a membrane using reduced pressure and temperature towards the target solution. Crystals are continuously removed from the feed solution by fractional precipitation. The process was optimized at a feed brine temperature of 50 °C and a temperature difference of 27 °C to minimize losses of Li-carnallite and to reach a 10 times faster evaporation [13].

The water-intensive mining process causes aquifer depletion and environmental degradation, which can be monitored from satellites by NIR and red light spectra reflected by vegetation. Increased water consumption is further due to tourism and population growth driven by the booming Li-industry, as well as climate warming. On site, temperature rises annually 0.74 °C in summer and 2.68 °C in winter due to increasing daytime temperature, whereas nighttime temperature remains constant [10].

Hard-rock lithium ores are dominated by granitic pegmatites. Explorations are focused on spodumene (LiAlSi_2_O_6_) ores, lepidolite (K(Li,Al)_3_[F(OH)_2_|(Si,Al)_4_O_10_]), petalite (LiAlSi_4_O_10_), lithium phosphates, and zinnwaldite contact zones. In volcanic clay deposits, lithium is either bound in structures of smectite and micas or adsorbed on clays [8].

Due to intrusions and hydrothermal fluid actions, lithium contents within the profile of a drilled core may vary widely at small distances, thus in prospection, averaging a larger distance is needed [8,14].

The Jiajika pegmatite-type rare metal deposit in the western Sichuan province in China is the largest spodumene deposit in Asia, but it also contains sulfide minerals. The mining area is located in the Jinchuan–Danba Li-Be-Pb-Zn-Au-muscovite mineralized belt, covered by quaternary sediments [15].

Extraction of Li from hard-rock ores is usually achieved by milling, mixing with sulfuric acid, roasting, precipitation of CaSO_4_ + MgSO_4_, and finally Li_2_CO_3_ precipitation with Na_2_CO_3_ [11].

Li-recovery from fly ash can be achieved by sodium salt roasting and organic acid leaching to form NaAlSi_3_O_8_ [14].

### 2.2. Mine Tailings and Pollution

Mining, smelting, and foundries are point sources for major Li contamination during the exploitation and processing of ore deposits, in particular from tailings, which often contain finely ground minerals [5].

In landfills or open dumps, Li gets easily leached and is rather mobile in subsurface layers. Lubricating greases used in vehicles manufactured from LiOH·H_2_O can release Li into the environment through surface-water runoff from roads. Urban runoff from Seoul city contributed significantly to Li inputs into the Han River [5].

In case ore bodies are covered with soil layers, soil extraction of mobile fractions can be helpful for prospection. If the ore vein contains sulfide minerals, groundwater level is high, and pH is sufficiently acidic, acid and metal salts migrate upwards mainly by capillary action. In the Jiajika area in the western Sichuan province in China, soil extraction methods of the <200 mesh (0.074 mm) fraction cored at 5–10 m and 10–20 m over the ore body, with water or 0.2 M K_2_SO_4_, detected concealed lithium deposits successfully, and the response increased from about 100 m outside the ore body. Water extracted about 0.01% and K_2_SO_4_ about 0.1% of total Li, but the results were significantly correlated [15].

The orebody of the Whabouchi mine in Quebec (Canada) is a large and homogenous albite-spodumen (LiAlSi_2_O_6_)-pegmatite, hosted in amphibolite. The mine tailings contain mainly quartz and feldspars (preferably albite), together more than 75%, and still 0.53% Li, but almost no C and S. The washout of Li from these mine tailings was investigated in a 4-year outdoor field experiment and a column experiment in the laboratory in parallel. The field cell was a 21 m × 21 m truncated square pyramid, and the column was of 1 m length and 15 cm diameter. Both were filled with a 1 + 1 mixture of spodumene flotation tailings and <850 µm particles from ore screening. Whereas outdoors there was ambient climate, the column was watered once a month with 110 mm rain and left dry in between. Field K and Li concentrations as well as release rates were similar, and Li release was still 3–5 mg/L. Electrical conductivity and most element concentrations (except Ca) followed a general declining trend versus time. The main differences between field and lab experiments occurred with respect to Fe, which reached just 0.01 mg/L from the column but peaked up to 36 mg/L in the open due to sulfide mineral oxidation [16].

In urban biowaste from four Austrian provincial cities, sampled monthly throughout a year, Li was also rather low but had not been measured in all samples. Mn was quite low, and just three samples from position 4 had more. Mn is usually strongly enriched in tree barks, and obviously the input of barks was low. Sr was rather uniformly distributed among biowaste and organic fertilizer samples, but less than expected in soils [17].

During the present day, reports indicate rapidly increasing Li in rivers and tap waters due to inefficient treatment. Lithium is not removed from drinking water with current treatment technologies [4]. In industrialized areas like Copsa Mica (Romania) associated with the battery industry, groundwater contained 148 µg/L, and some waste leachates in the US already reached 19,000 µg/L [18].

Remediation of Li-contaminated sites can be achieved by fixation of mobile amounts in zeolites and clay minerals as well as coal fly ash. The phosphate is only moderately soluble under neutral to alkaline conditions (378 mg/L for pure Li_3_PO_4_). Accumulator plants are, e.g., *Apocynum venetum* and *Apocynum pictum* [5].

Horizontal subsurface flow of 0.1 L/s through a constructed wetland with common reeds (*Phragmites australis*, 5 m^2^) grown on gravel 1–2 cm removed about 44% of Li from municipal wastewater of 320 mg/L COD during the summer season by wetland plant uptake. This was much less than for Cu and Zn, but more than for Rb, Sr, Co, and Ni [19].

Lithium emissions into the atmosphere may happen via dust particles from waste incineration and ore processing [5].

### 2.3. Recovery from Li-Ion Batteries

Anthropogenic use of Li-based compounds occurs in aluminum processing, pharmaceuticals, lubricants, Li-ion batteries, and glass and ceramic production. More than 80% of the total Li market will be used to produce Li-ion batteries, which currently have the worst recycling recovery rate among technically used metals [5]. From 2000–2012, global Li production tripled, driven by the use of Li batteries in electronic goods and vehicles [20]. By 2030, 11 million tons of spent Li batteries have been predicted [21]. Europe has the highest rate of collected and recycled e-waste at 42.5%; it is much lower in other parts of the globe [22]. Li is also contained in ceramics and mood-stabilizing drugs and at 1% in vehicular grease, with the latter leading to an increase of Li in run-offs from roads [5].

Lithium-ion batteries as efficient high-density energy storage cause high demands for global production. In South America, 70% of global Li reserves are concentrated, and Chile took up an average of 38% worldwide Li production in the past 20 years [10]. In the next decade, the demand for Li-ion-batteries for electric-powered vehicles is expected to demand globally a Li_2_CO_3_ equivalent of 2.4 million metric tons [9]. Spent Li-batteries contain 5–7% Li, which is higher than the amount in natural ores [23].

A Li-battery contains Cu-contact, a porous anode made of graphite, a porous separator made of polypropylene or polyethylene, a porous cathode, a polymeric binder like polyvinylidene fluoride, and an Al-foil collector. Common cathode materials are mainly LiCoO_2_ and less frequently LiMn_2_O_4_, LiNiCoMnO_2_, LiNiCoAlO_2_, and LiFePO_4_. Thus, spent lithium-ion batteries contain 5–20% Co, 5–7% Li, and 5–7% Ni, which are higher concentrations than in naturally occurring ores. The electrodes and the separator are soaked in an electrolyte consisting of 1 M LiPF_6_ in an organic solvent mixture. In the case of cars, the Li-batteries still retain a significant recharge capacity of 70–80% when they are removed from a vehicle and can still be used for storage of electricity in home energy storage systems. In case of short circuit or damage, there is fire risk, and the battery burns without external oxygen; thus, it has to be burned out. Spent batteries often retain a residual voltage, which can pose a safety risk of combustion or explosion. Therefore, it is typical to discharge, disassemble, and sort to recover the valuable black cathodic mass [21,22,23].

When the lithium-nickel-cobalt-manganese oxide cathode material samples are subjected to normal atmospheric conditions, secondary phases like LiMn_3_O_4_, LiMn_2_O_4_ and Li-free MnO, Mn_3_O_4_, NiOOH, CoO, and CoNiO_2_ are formed. These secondary phases are easily mobilized and thus are sources of metal release into surface water and soil [24].

In pyrometallurgical treatment of the entire spent battery, the organic component is at first thermally degraded, followed by the production of metal alloys using reductive reagents at around 1500 °C. This causes emission of HF from LiPF6 and the binder, as well as the formations of volatile organic compounds, which have to be cleaned from the exhaustion gases. Whereas Li and Al are usually difficult to recover this way, because they end up in the slag, the graphite of the anodic material causes additional heating, which leads to Li and Co metal at 1000–1200 °C. The high energy consumption of this process is disadvantageous [21,22]. The starting mechanical treatment is preferably done under N_2_ or under CO_2_ to prevent the build-up of flammable gases. Recovery of the electrolyte is possible by extraction with supercritical CO_2_ [21].

After mechanical crushing and separation, the valuable metals (LiCoO_2_, but also Cu, Al, Fe, and Ni) can be recovered by acid leaching with HNO_3_, H_3_PO_4_, HCl, H_2_SO_4_, and organic acids like malic, oxalic, citric, and formic acids at rather low energy consumption, but leaving large amounts of wastewater. After precipitation of CuS or Cu_2_S with SO_2_, Fe, and Al with NaOH and solvent extraction of Co, Li is left in solution and precipitated with Na_2_CO_3_. In particular for LiCoO_2_, use of 1 M oxalic acid is superior to HCl, H_2_SO_4_, or HNO_3_, because Co oxalate is precipitated (solubility 2 mg/L) and easily recovered by filtration, unless there is oxalate excess to form soluble Co-hydrogen-oxalate. The molar proportion of H_2_C_2_O_4_ and LiCoO_2_ should therefore be less than 2. In addition, Al and Fe remain in solution as complexes. In a lab-model procedure, the parameters for the leaching process were optimized at 150 min retention time, 95 °C heating temperature, 15 g/L solid/liquid ration, and 400 rpm rotation rate. The precipitated CoC_2_O_4_ enters the fluid in case of intensive rotation instead of formation of surface coatings [25].

Recovery of Ni and Co from lithium ion battery effluents could be achieved by sorption in neutral and alkaline solutions upon a special biochar made from aloe vera, leaving Li in the liquid phase, with subsequent recovery as Li_2_CO_3_ after CO_2_ addition. A two-step procedure at 200 °C/16 h and 800 °C/3 h in the presence of Fe_2_(SO_4_)_3_ led to formation of magnetite, which easily enabled the recovery of the biochar from suspensions and more than five times of repeated use. Final recovery of Co and Ni from the biochar was obtained by desorption into 0.01 M HCl [26].

Bioleaching with *Aspergillus niger*, which produces organic acids, is slow because of toxic metal actions [22].

Environmental concerns from illegal waste deposition if Li-batteries arise from soil and water pollution with carcinogenic Co and Ni, fire hazards from residual electric loads, and PFAS, which have been used as fire retardants in older batteries [3]. Though recycling is highly favorable, pyrometallurgy leads to metalliferous slags and emission of hazardous gases, and hydrometallurgy causes acid metalliferous effluents [23,27].

## 3. Geology

### 3.1. Rocks

Coarse-grained, intrusive igneous rocks, pegmatites, and sedimentary rocks contain Li-bearing minerals, like spodumene, petalite, lepidolite, amblygonite, and eucryptite. Li enrichments may also be found in authigenic and detrital clays. Carbonates have low Li but high dissolution rates and thus availability thereof [5]. Si, K, and Na, together with Li, Rb, Cs, Tl, Be, B, rare earths, and Ge, are granitophilic elements [28].

In ferro-magnesian minerals, like olivine, pyroxene, biotite, chlorite, and cordierite, Li most commonly substitutes for Mg-Fe in octahedrically coordinated sites. They form the main hosts for Li in most rocks [29]. Jadarite LiNaSiB_3_O_7_(OH), which is only found in Serbia, has been formed via hydrothermal alteration of volcano-sedimentary clay-rich sequences [8].

In the Transbaikalia region in Russia, mean Li has been determined as 73 mg/kg in granite, 56 mg/kg in basalt, 25 mg/kg in diorite and gabbro, 5 mg/kg in carbonate rocks, and 34 mg/kg in sandstone. Biotite was identified as the main Li carrier, containing 220–524 mg/kg [30].

In Europe, the largest resources are associated with segments of the Variscan chain, and in the Archean-Proterozoic Ukrainian shield, but they are hardly exploited yet [8].

The Iberian Peninsula is home of Europe’s largest lithium deposit belt, located in the north of Portugal and center east of Spain. The aplite-pegmatitic vein field at Gonçalo (Portugal) contains lithium veins (lepidolite), stanniferous veins, and mixed veins within quartz, feldspars, and muscovite [6].

In Austria near Wolfsberg (Carinthia), resources of 6.3 × 10^6^ tons at an average of 1.17% Li_2_O have been explored due to spodumene pegmatites [8].

On behalf of Italy, Dini et al. [8] present a strategy to find geological facies and rocks that might contain Li enrichments and Li minerals. In the Alps and the Apennines, some local Li enrichments have been found, which are currently too small for profitable mining. Li enrichments occur in pegmatites at Elba Island, in F-bearing granites in Southern Sardinia, and in some thermal fluids of Tuscany, Latium, and Campania. Li shows high concentrations in argillite clayey sedimentary formations of the Northern Apennine Upper Cretaceous units. The wide Li content heterogeneity observed at the outcrop scale has been confirmed at microscale, where phyllosilicate-rich micro layers contain up to 700 mg/kg. Interaction with groundwater can lead to substantial concentrations in thermal waters [31] (see Section 5.2).

In Hungary, Li enrichments in bauxites have been reported [8].

In the Monchegorsk ultramafic layered complex, which is a paleoproterozoic intrusion of the Kola region (Russia), and mined for Ni, Cu, and platinum group elements, lithium content was low and obviously homogenously distributed, at 4.37 ± 1.49 (range 2.03–7.47) mg/kg [32].

In Namibia (southwest Africa), total Li in Huab pre-cambrian granites ranged within 10–30 mg/kg (mean 22 mg/kg), whereas in the Damara cambrian and mesozoic granites, about 100 mg/kg were found. A Li content of >100 mg/kg in granites may be a useful indicator of extreme fractionation. In ferromagnesian minerals, Li^+^ replaces Mg^2+^ and Fe^2+^, and it gets enriched in late igneous differentiates. Li/Mg tended to increase to high values in some of the young granites [28].

### 3.2. Sediments

In the area off Hokkaido and the Tohoku region in the north of **Japan**, 1406 marine sediments 0–3 cm and 798 stream sediments from adjacent terrestrial regions were analyzed after HF-HNO_3_-HClO_4_ digestion. Sediments in the study area include sand and clay materials deposited after the Pleistocene age. The surveyed terrestrial areas are covered extensively by Neogene-Quaternary volcanic rocks and sedimentary rocks. Only Li, like K, Be, Ba, Al, and U in coastal areas had a distribution resembling those in adjacent terrestrial areas (Table 1). In the case of Li, no regional effects nor particle size effects were noted [33].

Marine surface sediments 0–5 cm were sampled in the **Pacific** Ocean along a profile from Japan to Mexico at 65 points. Lithium was found slightly enriched in pelagic deep-sea red clays and increased slightly towards Mexico and California, where weathering products of granite layers carry some more lithium than the basic rocks of the andesite zone of Japan. Fe-Mn concretions in the muds of the Hawaiian zone contained Li 1.5–3-fold enriched contrary to K, Na, and Mg and reached a range of 36–120 mg/kg. It is suggested that Li can enter the structures of the Mn minerals, or it gets enriched by sorption (Table 2). The products of basalt weathering lowers the contents of Li-Rb-Cs in the precipitates at submarine hills [34].

At the Bulgarian coast of the **Black Sea**, in surface sediments 0–5 cm sampled at 20 stations between 0.1 and 85 km from the coast, lithium was relatively uniformly distributed in all marine zones. After digestion with HClO_4_: and HNO_3_ in a ratio of 4:1, lithium ranged within 11–35 mg/kg [35].

In the **Adriatic Sea**, Zadar harbor sediment (reverse aqua regia digest) contained 2.5–23.8 mg/kg of Li, and no trend of grain size versus depth size appeared. Soils adjacent to the shore done by the same method had 10.5–46.4 mg/kg [36].

The **Slovakian** survey of stream sediments covers 24,432 samples sieved <125 µm, after total digestion, and showed Li enrichments in the Carpathian Mountains at 31–70 mg/kg, due to claystones and sandstones in the Flysch belt and Paleozoic metamorphosed rocks in the crystalline units. The lowland and basins were lower than the median of 29 mg/kg, except some maybe man-made enrichments in the lower tracts of the Vah River [37]. The **Austrian** stream sediment survey covered 3526 samples <180 µm after total digestion and resulted in about 10 mg/kg in limestone Alps, 16–37 mg/kg in pre-alpine lowlands, some enrichments in the Carnian Alps, and some small hotspots in central alpine regions [38].

In reservoirs of the River **Danube** in Austria, neither grain size nor regional inputs seemed to affect the Li contents (Table 3), even before the establishment of full sewage treatment, contrary to Co, Cr, Ni, Pb, V, and Zn [39].

Within a 25 km segment of the **Labe** River between Kolin and Nymburk, sampled 3 m off the shore and 0–10 cm depth in 1991, Li content in the fraction <63 µm was 17 mg/kg, after aqua regia digestion. Like K, Mg, Mn, Zn, Co, and Pb, the Li increased with decreasing particle size. In sequential leaching, exchangeable and Fe/Mn- bound Li was very low (<1 mg/kg), 4–5 mg/kg were released in the oxidizable fraction, and major parts were found residual [40].

In sediment cores from three **lakes in Mecklenburg-Vorpommern**, northeast Germany, profiles could be dated by 14C until 14,000 years back and various input identified by pollen. The lithogenic elements Al and Li (total contents) increase at the beginning of the high medieval settlement (around 800 years ago). The curves run parallel with herbs and grasses as indicators of settlement activities, whereas before this time, the concentrations of both elements were very variable in the sediment profile. High Al and Li indicate soil erosion. Different settlement phases are expressed by high grass and herb values, whereas phases of minor human activities are shown by increasing amounts of trees and shrubs [41].

In China, the basin of the **Xijiang** River occupies 78% of the Pearl River total basin area and accounts for 64% of the Pearl River discharge. Limestones and dolomites cover 45% of the total basin area. Shales, sandstones, and siltstones are widely distributed in the Upper Reach, while quaternary fluvial sediments are abundant further downstream. In river water, Li ranges within 0.96–1.31 µg/L in the upper part and 0.47–0.81 µg/L in the lower. The δ^7^Li was +25.7‰ in the wet season and 18.1‰ in the dry season. Total Li in suspended sediment ranged within 44–66 mg/kg and had a δ^7^Li of −3.8 to −0.7‰, much lower than the river water. River bed and bank sediments, however, had lower Li concentrations and higher δ^7^Li than suspended sediments. From this, as well as from Na, Ca, Cl, and δ^7^Li in the adjacent rocks, it was concluded that Li contribution from evaporite dissolution to the contents in the river water was less than 2% in both seasons, and the contribution of carbonate weathering varied from 7 to 19% during the wet season and from 8 to 23% during the dry season. Main amounts were derived from silicate weathering [42].

Li/Ca along the axis of maximum growth of great scallop **shells** (*Pecten maximus*) collected in the bay of Brest (France) showed high interannual but low seasonal variability. During diatom blooms, additional Li gets trapped in the shell via digestive dissolution of the diatoms, whereas temperature within the range 8–18 °C is of weak influence. Thus, Li/Ca in shells may be used as a proxy for timing and magnitude of diatom blooms in coastal ecosystems [43].

### 3.3. Coal

In Chinese coalfields, some Li enrichments have been reported, e.g., in Jungar coalfield in Inner Mongolia at 116–264 mg/kg. At this location, weathered denudation of K-feldspar-granite in the north provides the main source of Li enrichment. Average concentrations in various coal seams range from 55 to 75 mg/kg and maxima from 135 to 270 mg/kg. Li in coals occurs mainly in accessory minerals like kaolinite and other clay minerals, boehmite, and chlorite. Whereas for prospection of coal electrical resistivity, density and natural gamma radiation are used, for prospection of Li in coal deposits, it is additionally necessary to pay attention to minerals by means of scanning electron microscopy, X-ray diffraction, and electron microprobe analysis. Coal for extracting Li should have more than 50 mg/kg [14].

Lithium in eight Argonne premium coals ranged within 2.8–29 mg/kg (median 6.8) and in respective ash after treatment at 525 °C, within 29–150 mg/kg (median 102) [44].

Lithium distribution in fly and bottom ashes might be similar during and after coal combustion, but in some cases, fly ash may contain higher concentrations [5]. Fly ash from combustion of South African coal contained Li at 390 ± 15 mg/kg. Collection at an electrostatic precipitator in conventional and in pulsed mode yielded the same result, contrary to an increase of, e.g., F, S, K, V, Ni, and Pb in the fly ash [45]. Fly ash from the Chonqing coal-fired power plant (Hebei, China) also contained rather high Li in its fly ash, largely bound to insoluble forms as non-magnetic particles like aluminosilicate glass (58%) and mullite + quartz (30%), enriched in the grain-size fraction 38–25 µm, and emanating from a Li-bearing montmorillonite accessory mineral [46].

### 3.4. Dust

At 121 sampling sites all over **Croatia** in 2010, moss samples were collected during summer and autumn 2010 at >300 m distance from main roads, >100 m distance from local roads, >200 m from villages, and >3 m from nearest tree canopies. Parts of *H. cupressiforme*, *P-schreberi*, *B. rectubulum*, and *H. sericeum* were taken, representing the last 3 years. A mean Li concentration of 0.74 mg/kg resp. a median of 0.55 mg/kg, from a range of 0.11–4.27 mg/kg, was found. Maxima appeared around Dubrovnik, Knin, Rijeka, Krapina, and Osijek. Factor analysis put Li together with Al-Cd-Cr-Fe into factor 1, which made 25% of the total variability and represents a mixed geogenic-anthropogenic association of elements [47].

Fifty-five naturally growing moss samples (*H. cupressiforme*) were also sampled all over **Albania** in August/September 2015 to assess atmospheric deposition of the last 3 years. Li at a median of 1.35 mg/kg dry weight (range 0.37–4.3) was slightly higher than in neighboring **Macedonia** with 0.79 mg/kg (range 0.32–3.51) and much higher than in **Norway** with 0.16 mg/kg. Higher levels were found in the southeast and in the northern part close to the village Burrel. Factor analysis put Li together with Fe, Al, V, Ba, and Sr into the first component, which indicates crustal material and soil dust as the origin [48].

Total annual atmospheric deposition in the east of **Austria** was estimated at 0.88 g/ha, sampled at the northern rather rural border of Vienna in 1997/98 [49].

In **roadside dust,** aqua regia detectable lithium does not seem to be enriched to date (sampling 2010 and 2012). In Budapest city, 13.9 mg/kg (range 5.5–21.7); Budapest suburbs, 13.1 mg/kg (range 12.0–15.8); Seoul, 29.5 mg/kg (range 25.0–43.0); and Raleigh (North Carolina, US), 11.5 mg/kg were found, which matches the lithogenic background [50,51].

Incineration of domestic e-waste will be an emerging source of atmospheric emission [5].

### 3.5. Li-Isotope Effects

Isotopic fractionation may be divided into kinetic and equilibrium isotope effects. In evaporation, the lighter isotope has a higher velocity and is preferentially transported across a phase boundary. The heavier isotope is preferentially partitioned into lower coordination environments [52].

The average Li concentration of the upper continental crust was estimated at 35 ± 11 mg/kg, with an average δ^7^Li of 0 ± 4‰. Close to the bulk silicate δ^7^Li are also ocean ridge basalts, brines, moon samples, and meteorites [29]. Because ^6^Li and ^7^Li differ in about 15% of mass, isotopic fractionation of up to 50% is possible in low temperature environments [52]. During chemical weathering, ^6^Li and ^7^Li are strongly fractionated, with ^6^Li being retained in the newly formed clay minerals and ^7^Li leaving in solution [42]. In smectite clay formation, ^7^Li preferably remains in solution, whilst ^6^Li is preferentially incorporated into the lower energy octahedral sites [52].

Negative δ^7^Li can be observed in mantle peridotites, the upper and lower continental crust, and river suspended loads. Positive δ^7^Li may appear in river dissolved loads, lake waters, groundwater, and hydrothermal springs [29] and is due to control by incorporation into secondary minerals. In addition, sorption on gibbsite at 22 °C prefers ^6^Li, and carbonates demonstrate significant isotopic fractionation relative to water. Lack of temperature-dependent isotope fractionation in carbonates may record δ^7^Li and may record ancient weathering processes. In an experimental study with a coral reef, no dependence of δ^7^Li on temperature, pH, pCO_2_, or coral microstructure was found, and thus coral δ^7^Li may provide a proxy of paleo δ^7^Li in seawater. Modern seawater is homogenous with respect to δ^7^Li at +34‰, whereas riverine inputs to the sea cover a wide range of +1 to +44‰ (global mean +23‰), and dissolved river loads range from −6 to +5‰ [29].

Because Li isotopes are not fractionated during vegetation uptake, the isotope proportion remains constant in vegetation cycling. The Li isotope composition in stratified sediments permits the reconstruction of paleo-water isotopic composition and weathering intensity. Low temperature favors the removal of Li from solution by authigenic secondary mineral formation, whereas high temperature increases primary silicate weathering [42].

In aqueous corrosion of Li-borosilicate glass powder (50.5% SiO_2_, 15.86% B_2_O_3_, 8.93% Li_2_O), at 40° and 90° up to 464 days, a steady state of Li/B was reached after 28 days at 90° and after 126 days at 40°. XRD showed formation of lithium silicate, lithium aluminum phyllosilicate, and magnesium aluminosilicate as crystalline secondary phases. The δ^7^Li appeared similar at both temperatures; it was initially low and increased from 6 h until the end of the experiment at 464 days, finally being higher than in the pristine glass. This means that initially the Li-isotopes were leached incongruently through diffuse processes, but then the δ^7^Li increased until 28 days due to precipitation of Li-bearing smectite and subsequently remained constant [52].

Li-isotope fractionation can also take place at elevated temperatures and high pressure in subsurface geological processes during interactions between silicate melt and water, but this is beyond the scope of this article (see review of [29]).

## 4. Agriculture

### 4.1. Fertilizers

NPK fertilizers utilized in the northeastern US contained total Li (HF-digest) at a median of 1.1 mg/kg (range < 0.1–2.1). In dairy manures, the respective median was 2.2 mg/kg (range 0.3–6.8), which is less than that found in soils [53].

In Austria, mineral fertilizers were extracted with 3 M HCl at the boiling water bath and filtered, the residue was dry ashed to oxidize the coatings of the pellets, and they were leached again. Four grams of the sample ended up in 100 mL digest. It can be assumed that the total amount gets recovered. Because all frequency distributions of Li-concentrations have been found as non-Gaussian, median and 5–95% ranges are given to indicate the most probable value. All final determinations were done in the ICP-OES at 670 nm versus approximately matrix-matched calibrants (Table 4). In neutral ammonium citrate extract of NPK fertilizers, due to a more ample weight-to-volume ratio, Li was below 1.5 mg/kg [49].

Li in fertilizers is lower than expected for soils. In fertilizers, (acid leachable) lithium was found to be uniformly low, about 1 mg/kg in mineral fertilizers and commercial organics and about 2 mg/kg in limes, dolomites, Ca-Mg-phosphates, rock phosphates, PKs, and NPKs. Unlike Na (see above), there was no enrichment in K-salts. No regional differences in Li concentrations were noted. Just composts were significantly higher than others, corresponding to soil levels. Median lithium input from composts along with 100 kg N was 70 g and about 100 times lower than from manures; thus, Li/N seems to be a criterion to discriminate composts from any other fertilizers. Li/P in composts and garden molds was smaller than the proportion for mean soil values (Li/P = 0.02) The Li input for 100 kg P was 1–3 g from mineral fertilizers, 27 g from manures, and about 350 g from composts. Li/P in composts and garden molds was smaller than the proportion for mean soil values (Li/P = 0.02) [55,56]. Recently sampled composts from the Vienna region contained a median of Li at 13.6 mg/kg (range 8.2–25.6) after aqua regia digestion, which is slightly lower than in soils (Sager, unpublished).

Median concentrations of organic soil amendments in dry weight of animal origin were not entirely different: cattle manure, 3.2 mg/kg; pig manure, 3.5; pig dung, 1.4; and poultry dung, 2.1. Some higher levels occurred in biogas manure (7.2 mg/kg), compost (15.9 mg), and sewage sludge (33.3 mg/kg) [56].

### 4.2. Soil

Topsoil has lower Li contents than underlaying layers, and clay fractions have higher Li than organic fractions. Arid and saline soils generally contain higher background soil Li, due to a general enrichment of alkali metals. Clay minerals concentrate Li not only among exchangeables but also through isomorphous substitution at tetrahedral and octahedral sites. In general, Li adsorption in soils increases with increasing pH and decreasing ionic strength [5].

In **New Zealand** soils, aqua regia soluble Li was positively correlated with the clay fraction, as well as with Mg, B, Fe, K, Mn, and Zn. Soil and pasture samples of predominantly ryegrass were sampled to test the Li uptake versus aqua regia contents. Pasture grass Li was strongly positively correlated with Al and Fe and negatively with Mg, but not with any other soil properties. The average plant/soil concentration quotient was just 0.14 [20].

In 127 surface soils sampled all over **Poland**, sieved < 2 mm, and digested with HF/aqua regia/HClO_4_, a geometric mean of 11.5 mg/kg for Li was found. Only negligible differences were found between silty and loamy soils. The greatest proportion of variance was due to soil textural classes, which derive from soil parent material [57].

In 514 soils sampled throughout **Japan**, Li was determined after total digestion by ICP-MS at mass 7. The ranges were quite ample, with medians for andosols at 21 mg/kg, for cambisols at 45 mg/kg, for gleysols at 38 mg/kg, and for acrisols at 31 mg/kg. Slight Li maxima appeared at 25 cm depth [58].

A clay soil containing 39% clay, 29% CaCO_3_, and 1.26% organic matter sorbed Li at maximum from solution pH 4, at 85%. Removal of adsorbed Li by addition of 10% eggshells was optimized in the presence of acetic acid at pH 4 to obtain 41% sorption at 47.5 °C. The amount adsorbed at equilibrium was best fitted with a pseudo-second order equation [59].

In four types of forest soil profiles from Southern Carinthia (**Austria**), Li, Na, and K were quite immobile and had to be assigned to the residual fraction in sequential leaching. Whereas Li in aqua regia ranged within 9.6–39.3 mg/kg, 0.16 M acetic acid (1:20) leached only 0.01–0.08 mg/kg). The proportion of acetic acid-leachable Al-Be-Ca-K-Li-Mg-Mn over organic carbon increased with depth in all cores [60]. Similarly, in apple orchard soils 0–20 cm from the east of Austria, 0.16 M acetic acid leached Li at a median of 0.204 mg/kg (range 0.037–0.62), which is largely below 1% of the total, and subsequent 0.2 M oxalate pH 3 leached 0.54 mg/kg (range 0.135–2.56). The same samples yielded a median of 35 mg/kg (range 29.6–52.6) total Li and 21.1 mg/kg (range 15.9–33.7) from KClO_3_-HNO_3_ digest, the latter being equivalent to aqua regia [61] (Table 5).

A mixed soil from the open-pit mining area at Gonçalo (**Portugal**) was extracted in a Soxhlet (1.14 kg + 1.4 L H_2_O) to simulate weathering and released Li at 290 µg/L after 24 days, 203 µg/L after 48 days, and no more Li after 125 days of treatment [6].

In soil columns, vertical migration of Li gets retarded, whereas Cl is regarded as a nonadsorbent tracer for water flow. When a pulse of 25 mg/L Li (as LiCl) was added to undisturbed soil columns and repeatedly washed down, however, Li and Cl appeared at the same time in the outflow, but the relative concentration of Li was lower. This was explained by rapid movement through preferential flow paths [65].

When the vertical migration of a pulse of nutrients NPKS was tested in a column experiment during 3 months in the dark, Li together with non-added Na, Ca, Mg, Sr, and Ba peak at the elution of one pore volume of water. The total load eluted by the 40 eluates (about sixfold annual precipitation), however, did not match with the load obtained from standard water extraction 1 + 9 in the same volume of water (four replicates each). From the gleyic chernozem, 68.6 ± 7.8 µg eluted from the columns and 20.8 µg from the batch, from the haplic chernozem, 18.8 ± 9.4 µg eluted from the column and 41.7 µg from the batch, and from the calcic chernozem, 22.7 ± 2.8 µg eluted from the column and 14.0 µg from the batch. This may reflect ion exchange versus added K, soil life, and sorption at secondary precipitates [66].

A quite concentrated solution of 0.4 M LiCl has been proposed as an extractant for mobile cations in soils, which hardly changes the soil pH and permits determination of Ca, Mg, Na, and K. Good correlations with CaCl_2_ and BaCl_2_-extractables were achieved, but the extracted amounts were lower due to its weaker exchange efficiency [67].

### 4.3. Plant Growth

In soils, Li is found primarily in the clay fraction. Lithium is taken up by all plants, although it appears not to be required for growth and development. Excess Li decreases plant growth and interrupts carbon assimilation, uptake of mineral elements, and the antioxidant system, but plants vary greatly in their capacity of Li tolerance. Li tolerance also depends upon available K and Ca because of competitive uptake. Oxidative damage is indicated by the malondialdehyde test [5,68]. At high levels, it reduces chlorophyll and carotinoid contents, causing chlorosis. It is relatively toxic to citrus plants, whereas nightshade species are tolerant accumulators. Significant symptoms can appear at >50 mg/kg in soil or nutrient solution [5], but halophilic plants like *Carduus arvense* and *Holoschoenus vulgaris* may tolerate rather high lithium contents of up to 1000 mg/kg [7].

Median concentration values obtained in a ring test issued by Wageningen Agricultural University were obtained for Li in oak at 145 µg/kg, in wheat grains at 152 µg/kg, in grass at 964 µg/kg, and in tobacco leaves at 7493 µg/kg [69].

In green leaves, Li concentration tends to increase towards senescence, like, e.g., lanthanides. Thus, juvenile beech leaves in Southern Sweden contained 42 µg/kg; in September, 48 µg/kg; in November, 76 µg/kg; and in the leaf litter, 266 µg/kg [70].

In organic soil layers, mass loss during decomposition of leaves and branches is expected to cause concentration increases of lithophilic elements like lithium, unless washout dominates. When leaves or branches of Korean red pine (*Pinus densiflora*) and Korean chestnut (*Castanea crenata*) were packed in litter bags of 2 mm pore size and put to forest soil for one year to undergo natural decomposition, lithium in pine needles increased from 0.23 to 1.00 mg/kg, in chestnut leaves from 0.25 to 12.6 mg/kg, and in chestnut branches from 0.10 to 0.64 mg/kg. However, Li decreased in Korean pine branches from 0.68 to 0.45 mg/kg [71].

In the Transbaikal region of Russia, which is rich in Li in soil forming rocks, the mean Li content in the samples of steppe vegetation was found at 1.8–2.0 mg/kg dry mass (range 1.2–2.6), and for meadows, it was 3.2–4.0 mg/kg (range 1.4–8.9), because in the steppe, the productivity is 7.7–8.0 times lower than that of meadows and cultivated vegetation [30].

A silt loam soil of 31.8 mg/kg Li was amended up to 100 mg/kg Li to test the tolerance of green plants during 3 months of growth. This led to Li uptake into the leaves up to 1000 mg/kg without biomass reduction, but Li in the bulbs of *Beta vulgaris* was 10-fold lower. Whereas *Lactuca sativa* and *Beta vulgaris* leaves showed positive correlations between Li and Mg, leaves of *Lolium perenne* were negative. In *Helianthus annuus*, maximum Li uptake occurred in the bottom leaves, accumulating 756 mg/kg and suffering from necrosis, but without change in biomass [20].

Whereas many green plants accumulate Li mainly in the roots or favor Li excretion, the Li-accumulator *Apocynum venetum* (leaf dogbane), a halophile of temperate climatic zone, translocates and accumulates Li in its leaves. Seedlings were raised hydroponically, and after 28 days they were exposed to Li at 0, 2.5, or 25 mg/L. After 28 days of further hydroponical stay, the plant was separated into leaves, stems, and roots and investigated to detect subcellular sites of Li uptake. Most of the additional load moved to the soluble fraction, and the least moved to the mitochondria. Sequential leaching of the plant tissue (1:20) with 80% ethanol—water—1 M NaCl (for pectinates and protein-integrated Li)—2% acetic acid (for Li-phosphate)—0.6 M HCl—residue was performed to indicate Li speciation. At a high Li level (25 mg/L), major parts of additional Li were extractable with 80% ethanol (range 65–92%), increasing in the order roots < stems < leaves, while other fractions remained almost unchanged. Thus, the high tolerance can be explained by ligand binding in the vacuole [68].

In irrigation water for plant growth, a maximum Li-concentration of 2500 µg/L has been recommended [72].

### 4.4. Commercially Available Feedstuffs

Table 6 and Table 7 contain data obtained from routine control of commercially available feedstuffs, which are in the market for farms feeding more animals than they supply from their own area. Lithium levels in commercial feedstuffs are substantially higher than in human nutrition (see Section 7), even if differences between dry weight and wet weight are considered, contrary to the basic food items silage and leaves in Table 6. Supplementary feeds contain almost double the Li compared to composite feeds [73,74] (Table 7). Commercial feedstuffs contain basic feeds plus additions of trace elements, vitamins, antibiotics, appetizers, etc. In most trace element preparations analyzed, lithium was below 0.2 mg/kg, but some contained higher levels due to an Fe-Mg-Mn component, and the maximum was 22.6 mg/kg; however, Fe-only preparations were low (unpublished results).

Stomachs of wild ducks from four separated regions of Austria contained Li at a median of 1.11 mg/kg (range 0.12–5.64), but in corresponding tissues only 0.008 mg/kg (range 0.003–0.233) dry weight, without significant correlation [75,76].

## 5. Water

### 5.1. Limnic and Potable Waters

Typical background concentrations of Li in surface water range within 1–10 µg/L but can reach up to 500 µg/L in groundwater, due to weathering of Li minerals or saline intrusions. Its occurrence is correlated with other alkali metals, particularly Na [5]. Li/Na, Li/Mg, Li/K, and Li/Ca ratios in ambient waters can provide an archive of previous environmental conditions, like temperature, salinity, clay formation, and pH [18].

In order to obtain a fingerprint via the proportion of concentrations found in a given water sample over reference values, a reference freshwater dataset has been established from the mean of multi-element datasets, excluding extreme values of geogenic or anthropogenic origin. For the alkali metals, reference concentrations have been set as follows: Li = 3 µg/L, Na = 5000 µg/L, K = 2000 µg/L, Rb = 1 µg/L, and Cs = 0.05 µg/L [77].

In European tap water, median Li is at 2.65 µg/L, and in bottled water, a median Li at 14.8 µg/L was estimated, but large fluctuations attributable to lithology and geology occur [78]. A short overview is given in Table 8.

Because lithium from water is highly bioavailable, a screening of drinking and mineral water is necessary to obtain possible effects on human health due to systematic exposure. It may be accumulated in the thyroid gland [18].

In Sogndal (southwestern **Norway**), the mobility of LiF in a catchment of a small lake was monitored by spraying LiF-enriched lakewater to the catchment. The location is characterized by alpine vegetation and acidic soils on siliceous gneissic bedrock. Li as a mobile cation should provide a tracer for the applied water, but strong retardation occurred. After the start of the experiment, pH increased by two steps, first from 5.40 to 5.64 when discharge started and further to 5.91 after 48 h. Lithium and fluoride followed the same timing, with a maximum at 8 h 45 min, but only 1.62% of added Li (1.09 g out of 67 g) and 0.2% of added F left the catchment. LiF addition caused slight decreases in H, Ca, Mg, Na, and K and increases in Al and Li in the runoff water, whereas in a parallel experiment with NaCl, H, Ca, Mg, Na, K, and Al increased. Unlike fluoride, Li did not participate in any of the key processes affecting Al mobilization [89].

In **Denmark**, drinking water supply is entirely of groundwater origin and highly decentralized. Aquifers are mainly unconsolidated quaternary glacial sand, tertiary marine and fluvial sand, or cretaceous limestone and chalk. Common treatments are only aeration to remove CH_4_, and sand filtration. In a survey of 139 locations sampled at exit waterworks and filtered at 0.45 µm, a median of 10.3 µg/L (range < 5–30.7) was found. Lowest concentrations were met in western and northern Jylland and increased towards the east, with maxima in parts of Sjaelland, Møn, Falster, and Lolland. Because of low population density in respective areas, only 1.8% of the Danish population gets exposed to Li > 25 µg/L. Sampling sets from earlier times indicate that Li concentrations are stable over larger time scales [79].

In **Latvia**, 720 groundwater wells were sampled throughout the country, mainly until 10 m depth, filtered at 0.45 µm, and analyzed by ICP-MS. For Li, a median concentration of 2 µg/L was obtained, without significant influence of pH and redox potential. Just 10% of the samples contained more than 10 µg/L. In detail, median Li in shallow wells was found at 1.3 µg/L, in springs, it was found at 1.9 µg/L, and in shallow wells in confined aquifers, it was found at 5.5 µg/L. Concentrations of Li, Sr, Ba, and Fe got higher with increasing residence time and increasing confinement of the aquifer. Principal component analysis assigned Li together with sulfate, Ca, F, and Sr. This could be interpreted as a factor due to dissolution of gypsum containing carbonate rocks or of influx of more saline groundwater from adjacent aquifers. Elevated concentrations of F and Sr typically occurred in Ca-saline groundwater, but because fluoride concentrations did not exceed 1.5 mg/L, there was no control from the solubility of CaF_2_ [80].

In a peatland catchment in the Jizera Mountains (**Czech** Republic), formed by Paleozoic crystalline bedrock overlaid by shallow highly permeable cambisol, lithium contents fluctuate significantly between the precipitation (0.35 ± 0.21 µg/L), the shallow ground water (1.87 ± 0.23 µg/L), and the outflow (3.10 ± 0.36 µg/L). There is no link between the Li contents and the rainfall amount, but a positive trend can be seen between Li contents and the outflow discharge. Li isotopes data show an increase in the δ^7^Li between the precipitation (+5.6 ± 2.7‰), the shallow groundwater (+13.9 ± 4.4‰), and the outflow (+18.4 ± 3.5‰) [84].

In addition, the mean Li level in water samples from drinking water supplies taken from 99 **Austrian** districts was 11.3 µg/L. The highest district mean level appeared in Mistelbach at 82.3 µg/L, and the top single point was at 1300 µg/L in the vicinity of Graz [82].

The chemical characteristics of the River Danube remain rather constant in the Austrian part, with respect to pH, hardness, and conductivity. Whereas the concentrations of dissolved matter get lower with increasing water level, Li and K, however, increase [87,90] from 2–5.6 µg/L to 7–12 µg/L at high flood.

In **Switzerland**, groundwater monitoring at 44 sites resulted in a median of 3 µg/L (range 1–7, max. 11) [91].

In the peat bog of a maar depression in the Massif Central (**France**), Li contents fluctuate significantly in the groundwaters, ranging from 0.07 µg/L in springs feeding the peat bog up to 195 µg/L in the groundwater. δ^7^Li are extremely variable within the site, ranging from +12‰ in the stream draining the area up to a δ^7^Li-rich value of +1226‰ in groundwater from the peat bog, only explainable by an external input due to Ca-amendment, used in local agriculture [85].

Across 47 subdivisions of the east of **England**, levels in drinking water ranged between <1 and 21 µg/L [83].

In **Portugal**, Li in the public water supply is 10 µg/L at the median, but a maximum of 191 µg/L has been reported. Close to the Li open pit mine at Gonçalo, surface water contained 8.7–19.8 µg/L, springwater had 25.6–35.4 µg/L and groundwater had 6.9–74.1 µg/L, which is less than in some mineral waters far from mining areas [6].

In **Greece**, a screening of 149 drinking water samples from both urban and rural areas resulted in an average of 11.1 µg/L (range 0.1–121 µg/L). The highest level was found on Samos Island, and the lowest was found on Chios Island. Twenty-one samples of commercially available bottled water contained a mean of 6.2 µg/L [81].

The average Li concentrations from public wells in **Texas** counties as results from 3123 water samples ranged within 2.8 and 219 µg/L, probably due to higher salinity and dry climate [86].

In San Antonio de los Cobres and surrounding Andean villages in northwestern **Argentina** in the Puna highland, Li up to 1000 µg/L has been found, but also high As, B, Rb, and Cs. In this arid highland surrounded by volcanic bedrock, Li, Rb, Cs, and Ba concentrations show similar patterns, likely due to geothermal activity and salt deposits. All elements showed only small temporal variations. In parallel to respective water supplies, the urine of 200 local women was analyzed, and some correlation between water intake and urine was established (r = 0.173/*p* = 0.029). At the location of 1003 µg/L in drinking water, Li in urine ranged between 852 and 14,300 (median 4550) µg/L, and at the location of lowest Li, urine ranged within 117–2220 µg/L. This means that high Li in water largely influences Li uptake and excretion, but there are additional effects due to locally grown vegetables [88].

### 5.2. Bottled Water, Mineral Water, and Thermal Springs (Table 9)

The lethal concentration of dissolved Li for human ingestion is 5 g/L [92]. Concentrations > 15 µg/L in drinking water are protective against dementia [18]. Except in locations of salt brines, slightly increasing Li content in potable water seems to decrease suicide rates [18]. Possible beneficial medical effects should appear within 50–100 µg/L [93]. Usually, Li concentrations are not labeled on the bottle and are thus hardly traceable for consumers.

In Germany, Li in bottled water ranged 0.51–2790 µg/L, and in tap water, it was 0.27–74.9 µg/L. Minima in well water were found in Northern German Lowland, and maxima were in Hesse and the Rhine-Eifel region. The highest Li contents were in saline fossil water due to the decomposition of mica. Storage in PET, green glass, or clear glass did not change its Li concentrations [93].

In the hot springs of Bad Gastein/Salzburg/**Austria**, mixed from 17 wells, Li occurrence of 186 µg/L had been found to be rather high. These waters near the Alpine main ridge are known for their radon content (20 nCi/L in the mix) and to cure rheumatically chronical diseases, besides high silica (50 mg/L) and fluoride, but the main ions are Na_2_SO_4_ [94].

**Table 9:** Li in bottled and thermal waters (µg/L); qu = quartiles.

Location	Mean	Median/Range	Remarks	Reference
Metamorphic rocks (I)		1.35		[95]
Plutonic rocks (I)		3.66		[95]
Sedimentary rocks (I)		3.84		[95]
Sedimentary metamorphic rocks		1.3		[95]
Sedimentary-volcanic rocks (I)		1.69		[95]
Volcanic rocks (I)		17.1	Max 241	[95]
Tolentino (I)	370,000		Thermal water	[8]
Salsomaggiore (I)	96,000		Thermal water	[8]
Castrocaro (I)	80,000		Thermal water	[8]
Montecatini town (I)	5000		Thermal water	[8]
Campi flegrei (I)	>10,000		Thermal water	[8]
Siena (I)	20,000		Thermal water	[8]
Ischia Island (I)	41,000		Thermal water	[8]
Veii (I)		442/517/544	Thermal water	[96]
Romania	4.8	qu 0.40–94	Mineral water	[18]
		qu 0.48–7.0	Drink water	[18]
Slanic Moldova (Ro)		29–1390	Mineral water	[97]
Greece < 4 g/L		76.2/0.79–1625	Thermal water	[98]
Greece > 4 g/L		1333/104–17,630	Thermal water	[98]
Greece cold gas-rich		22.3/0.77–5683	Thermal water	[98]
Greece acidic		66.2/9.65–11,278	Thermal water	[98]
Greece hyperalkaline		0.38/0.09–41.4	Thermal water	[98]
Greece	6.2		Drink water	[98]
Bad Gastein (A)	198		Mineral water	[94]
Austria		0.9/0.1–45.8	Well water	unpublished
Germany		31.4/0.51–2790	Bottled water	[93]
Germany		0.27–74.9	Tap water	[93]
Croatia		0.17–880	Bottled water	[99]
Serbia		43.5/0.76–985	Mineral water	[100]

In **Italy**, among 158 samples of bottled commercial waters, the effect of local geology has been outlined. Lithium showed higher concentrations in association with alkalic volcanoes and from interaction with evaporites. Factor analysis of all sample data revealed closer relation of Li with a factor dominated by Cl, Br, Na, and Si and much less with a factor dominated by Ca, Mg, carbonate, and sulfate [95]. In **Italy**, 60 thermal waters have been listed to contain >1 mg/L, and the top value is 370 mg/L at Tolentino in the province of Macerata. To give some further examples, a value of 96 mg/L occurs at Salsomaggiore (prov. Parma) and Monticelli Terme (prov. Parma), with 80 mg/L at Castrocaro (prov. Forli), 5 mg/L at Montecatini town (in Tuscany), 20 mg/L at Siena, up to 10 mg/L at Campi Flegrei (in Campania), and 41 mg/L at Ischia Island [8].

The thermal springs at Veii (16 km northwest of Rome’s center) were used as sacred water for healing purposes during the Etruscan until Roman Imperial times. Hot mineralized groundwater from deep carbonate aquifers and cold groundwater from a shallow volcanic aquifer are mixed to yield todays hypothermal springs of high Li level (442/517/544 µg/L, filtered 0.45 µm) but also with high B, Sr, F, Fe, and Mn. The discharge rate continuously decreased in historic times down to 1 L/s by self-sealing of the feeding fractures due to mineral precipitation and argillification of silica minerals [96].

Li in 14 **Croatian** bottled water samples was highly variable and ranged between 0.17 and 880 µg/L. Median concentrations were 0.29 µg/L in the Dinaric and 3.15 µg/L in the Pannonian region [99]. The effect of bottle material upon adsorption and release of trace elements, PET, and PAHs within 2 days after filling was also investigated for artesian bottled mineral water from artesian wells in western **Croatia**. In the case of the Li-low-level spring from Triassic dolomite rocks with 3.6 µg/L at the source, Li in glass and PET increased insignificantly, whereas no significant changes at the Li-high level spring from basic volcanic rocks at 834 µg/L occurred [78].

In 13 bottles of selected mineral waters from **Serbia**, Li significantly correlated with Na, K, B, carbonate, and sulfate, but not with Cl, Mg, or Ca. Higher Cs, Li, Rb, and Sb indicated water sources from granite intrusions [100]. Seven spa mineral waters from Vrnjačka Banja contain Li within a wide range (29–990 µg/L), because they drain from different aquifers, like Paleozoic schists, marble, serpentinite, sandstone, and breccias. Li strongly correlated with Na, K, Ca, Sr, and Ba, but not with Mg, Si, and sulfate [101].

Bottled commercially available mineral waters and spring waters from pastures were collected and analyzed without filtration in order to establish a database of Li, Na, Mg, K, and Ca in consumable waters in **Romania**. All analytes were non-normally distributed, and no contaminations from glass or PET (polyethylene terephthalate) were noted. Contents in water reflect that Li is enriched in saline-type deposits, shales, and granitic rocks versus carbonates and frequently associated with volcanic activity, resulting in some high flyers among Li concentrations. Approved bottled mineral waters contained Li at a median of 4.8 µg/L (quartile range 0.40–94), and 41% of them exceeded the recommendation of 10 µg/L (=non-regulatory health-based screening level). Other bottled water contained Li at a median of 2.1 µg/L (quartile range 0.48–7.0), and the springs contained 1.7 µg/L (quartile range 0.71–5.8 µg/L). Correlations between Li and Mg (r = 0.98) as well as Li and Na (r = 0.68) in spring water were highly significant, but also between Li and Na-K-Mg in mineral water. Groundwater in lowland Romania contains Li at 1.4–12 µg/L [18].

Natural mineral waters from the spa resort Slanic Moldova in Bacau County (Romania) have attractive therapeutic properties due to high Li and Mn properties. Four springs are alkaline of NaK-HCO_3_-Cl mixed type at pH 6.3 and contain Li up to 1390 µg/L and Mn up to 1103 µg/L. Two additional springs of alkaline bicarbonate type contain Li at 119 and 54 µg/L, and an additional spring of Ca-Mg-Na-K-SO_4_ contains Li at only 29.4 µg/L. Li concentrations correlate well with Na (r = 0.9690), Mg (r = 0.9687), and Mn (r = 0.9597) [97].

In **Greece**, some thermal waters contain extremely high Li, up to 17.6 mg/L. Li is governed by four important factors, which are salinity, which depends on seawater intrusion and water-rock interaction; on acidity; on weathering of ophiolitic rocks; and on hydrothermal activity. Factor analysis of all 276 data put Li together with all main components into factor 1. The Li/Cl ratio can be up to three orders of magnitude higher than that of seawater, thus indicating a strong water-rock interaction. No newly formed Li-containing minerals were found within the hydrothermal system [98].

In South Africa, Li in commercial bottled drinking water (21 samples) was found at 2.0 ± 0.03 µg/L [102].

In Semi and Dazi geothermal fields at the Southern Qinghai-Xizang plateau (Tibet), hot springs of extraordinary non-haline Li and Cs concentrations appeared, exceeding 35 mg/L and 50 mg/L, respectively. These boiling springs of pH 7.85–9.15 form fumaroles and steam and are supposed to react with host rocks of granite gneiss intruded by leucogranite and pegmatite, at 200–235 °C at subsurface positions. Due to the low boiling point of 86 °C because of the altitude at 4200 m, an enrichment of 9 mg/L was estimated from steam loss and δ^18^O values. Low Rb compared to high Li and Cs was explained by preferential sorption of Rb on clay minerals along fluid pathways. Robust positive correlations between dissolved Li and Cs and Li and Cl were found [103].

### 5.3. Seawater

In oceans, the dissolved fraction of 170–180 µg/L is homogenously distributed throughout the water column and from tropics to polar regions. It is, however, enriched in brine deposits [5]. In the Ria de Aveiro coastal lagoon, ambient Li of 203 µg/L was found [104].

Within a 10 km wide strip of land parallel to the coast, 104 water samples from groundwater wells and surface water in and around Ravenna contained a median Li of 45 µg/L but ranged from 3–180 µg/L, the level of the Adriatic Sea. This southeastern Po River floodplain is a low-lying coastal aquifer that is affected by salinization from groundwater over-exploitation, drainage, and sea-level rise [72].

## 6. Biochemistry and Toxicity

### 6.1. Molecular Biology

Lithium ions do not bind to proteins but change the shape of phospholipids located in the outer and inner monolayers of the human erythrocyte membranes. Li effects upon changes in the structures of dimyristoylphosphatidylethanolamine and dimyristoylphosphatidylcholine were monitored by X-ray diffraction in glass capillaries, and also some hydrophobicity changes were monitored by fluorescence spectroscopy of interactions with 1,6 diphenyl-1,3,5-hexatriene and 6-dodecanoyl-2-dimethylaminonaphtalene. Li^+^ produced significant perturbation, particularly to the lipid polar group. The phospholipid acyl chain was disordered at 18 °C (Li-range 7–70 mg/L), but at 37 °C, it got more ordered at 7 mg/L and disordered at higher Li again. Li probably interacts with the phosphate and carboxylate groups of choline and serine [105].

After incubation of erythrocytes in phosphate-buffered saline with pH 4, fixation in glutaraldehyde, and gold coating, morphological changes were visualized by scanning electron microscopy. Cell shapes of the echynocytosis type appeared, showing jagged cytoplasmatic notches as artefacts, which indicate cell membrane disorders [105].

### 6.2. Animals

For aquatic organisms, effects upon growth and reproduction get lower with increasing Na concentration [5,92]. The concentration of 50% lethality LC_50_ varies widely, from *Dreissena polymorpha* (zebra mussel) at about 200 mg/L until *Oncorhynchus mykiss* (rainbow trout) at 0.6 mg/L [104].

Mussels *Mytilus galloprovincialis* were taken from the Ria de Aveiro coastal lagoon of ambient Li 203 µg/L and raised in artificial seawater and spiked with 0/250/500/750 µg/L of Li. Li additions increased liquid peroxidation levels and decreased glycogen as well as the bioconcentration factor, which may be related with efforts to avoid accumulation of Li at higher exposure. Lower aerobic respiration, indicated by the ratio of reduced/oxidized glutathione and acetylcholinesterase activity, may be interpreted as a defense behavior, such as valve closure and reduced filtration rate [104].

Temperature rise from 15° to 21 °C and increase in Li from ambient to 555 µg/L upon the movement and feeding behavior of the mussel-carrion feeding **snail** *Tritia neritea* were investigated to predict possible pollution and climate effects. This snail lives on intertidal marine sandy beaches and feeds on mussel carrions. In model aquaria, preys of *Solen marginatus* and *Mytilus galloprovincialis* were set at a 6 cm distance to note the time spent stationary, moving and not foraging, searching, and feeding. Though *Mytilus galloprovincialis* did not occur in its natural habitat, *Tritia neritea* did not show any preference. Both temperature rise and Li addition lowered the survival after 28 days and significantly reduced the foraging activity. At the higher temperature, finding the carrion was quicker, and food consumption under Li stress was lower [106].

LC_50_ for fish ranges from 13 mg/L until >100 mg/L; it is lowered during long-term stress [5]. In fish, lithium uptake happens most likely via a putative Na channel in the gill. When juvenile **rainbow trouts** (*Oncorhynchus mykiss*) were exposed to 66 µg/L for 9 days and subsequently to 528 µg/L for 6 days, length and body mass remained unchanged with respect to control water of 7 µg/L, at a Na/Li molar excess of 600. Li exposure declined plasma Na on the second day, but after this, it returned to pre-exposure values. The NaK-ATP-ase in the gills and free fatty acids got significantly reduced, but citrate synthase and plasma lipids remained unchanged [107].

When young **rainbow trouts** (*Oncorhynchus mykiss*) were kept for 21 days without feeding at ambient Li-K (<0.02 µg/L; 3 mg/L) versus elevated Li-K concentrations (36 mg/L; 115 mg/L), free fatty acids and cholesterol in the exposed gills fell sharply, and mitochondria numbers and liver weight decreased, but parasites (*Trichophyra piscium*, a ciliate) did not differ [92].

**Tadpoles** of *Rhinella arenarum*, which is one of the most abundant amphibia in the South American lithium triangle area, were used as test organisms to determine mortality rates of short-time and long-term lithium stress, as well for physiological alterations. They were kept in an aqueous solution of pH 8.1, 83 mg/L CaCO_3_, and 5.5 ± 1.5 mg/L O_2_ at 24 °C and fed with boiled lettuce. For evaluation of acute toxicity, they were exposed to up to 412 mg/L of LiCl. In the first 24 h, no tadpole mortality was observed, but then mortality rapidly increased, and LC_50_ was 320 mg/L after 48 h, 167 mg/L after 72 h, and 67 mg/L after 96 h of LiCl exposure. Within 2 weeks of chronic exposure at only 2.5 mg/L, enzyme activities of alkaline phosphatase, alanine aminotransferase, and aspartate aminotransferase as well as the level of the thyroid hormone T4 increased, and fecal pellet production doubled. At a 2-week exposure of 20 mg/L, these enzymes decreased, fecal pellet production increased sixfold, and higher micronuclei frequency and nuclear abnormalities in erythrocytes occurred, indicating a strong genotoxic effect. Diarrhea means loss of food energy [9].

In pastureland soils, **earthworms** *Eisenia fetida* usually contain Li at 0.55–1.20 mg/kg dry weight. After spiking soil with soluble Li_2_SO_4_, which was largely mobilizable by weak acids, they could accumulate until 106 mg/kg in their tissue, preferably (75–81%) in the cytosol, which was obtained after homogenization and centrifugation. The first histopathological changes were degeneration of seminal vesicles, indicating the decline in the reproductive capacity. Further changes included fibrosis of the body wall and intestinal muscle. Within 10–80 mg/kg additions, antioxidant enzyme activities increased, like superoxide dismutase, peroxidase, catalase, catalase, acetylcholinesterase, and glutathione-S-transferase, whereas metallothionein, 8-hydroxy-2-deoxyguanosine, and malondialdehyde decreased. No mortality occurred after 10 and 20 mg/kg addition after 7 days of exposure to 95 mg/kg, and after 1 day of exposure to 865 mg/kg, the LC_50_ was reached [108].

In order to investigate the molecular mechanism of lithium neuronal toxicity, male **mice** of 8–10 weeks age were orally administered a daily dose of LiCl for 28 days via drinking water, while the control mice were orally administered the equivalent dose of normal saline. After treatment, animal brains were harvested for analysis. For histological investigations, brain tissues were preserved in 4% paraformaldehyde, dehydrated, sliced, and dyed with hematoxylin-eosin. The LiCl-treated mice suffered from neuronal shrinking, neuronal degeneration, and apoptosis in the hippocampus, the area associated with learning, emotional processing, and memory. Apoptosis was made visible by binding dUTP-enzyme to paraffin-embedded tissue cells, and degenerated neurons could be stained by treatment with Fluoro-Jade solution. With respect to interaction between LiCl and proteins, 111 targets as well as target genes could be identified (details given). LiCl induced neurotoxicity by inducing apoptosis via the signaling pathways of MAPK and PI3K/AKT [109].

In order to provoke possible deficiency symptoms, **rats** were fed with a corn-casein diet of only 5–15 µg/kg, which is about 1/30 as the usual amount. Whereas in most tissues, a 20–50% reduction in lithium contents occurred, the Li-deficient rats retained Li at control levels in the pituitary and adrenal glands, and also in the bones. Conception time got prolonged.

Similarly, **goats** fed a low Li diet required repeated inseminations for conception an had reduced conception rate, increased barreness, and, in the case of pregnancy, double the probability of getting male kids. Effects of Li deficiency in goats were accompanied by atrophy of the spleen, lowered immunological status, chronic inflammations, and calcifications of the blood vessels. The newborn kids had lower birth weight and showed lower weight gains. Lithium deficiency in kids and goats reduced the activity of enzymes of the citrate cycle, as well mono-amine oxidase activity in the liver, but elevated creatine kinase [7].

Remarkable differences in tolerance towards LiCl has been proposed to kill the parasite **Varroa** mites at honey bees. When mite-infested honey bees were caged and fed with 174 mg/L Li as LiCl in sucrose syrup, 96% of the mites were killed within 3 days, without changing the survival rate of the bees. Mite mortality was recognized at <14 mg/L. The effect on the bees mortality, however, started at 10–15 days of permanent feeding with LiCl in sucrose syrup. Other Li salts were equally effective for the mites, but in the case of sulfate and acetate, worker bee mortality also slightly increased. Equimolar additions of NaCl, KCl, and MgCl_2_ to sucrose syrup had no effect. Whereas doubling to 348 mg/L had no further effect for caged bees, it is recommended for swarms of 20,000 bees to ensure adequate distribution [110].

### 6.3. Men

In blood serum (sampled in Rome/Italy), Li belonged to the group of elements occurring within the same concentration interval as adults and term or pre-term newborns, at 0.50 µg/L (range 0.2–1.8) [2].

Its deficiency causes no obvious symptoms in humans. At 15–20 mg/L blood concentration, it causes nausea, visual impairment, and kidney problems. Excretion occurs predominantly via the kidneys [5].

Lithium-containing preparations are leading in prophylactic treatment against manic depressive illnesses. Ingestion of lithium preparations should lead to a level of 5.6 mg/L in serum, initially controlled daily, later weekly. Blood sampling should take place 12 h after the last ingestion. A further increase in Li levels led to no improvement of results. Because of a reduced renal excretion rate, the dose for older patients should be limited to reach 4.2 mg/L. About 15% of patients do not respond to lithium therapy. Contraindicative to Li therapy are acute renal failure, cardiac infarction, irregular heart rhythm, malicious symptoms of the cerebellum, and feverish illness. In addition, Li treatment during the first trimester of pregnancy may lead to cardiovascular malformations of the fetus and thus should be omitted. Among initial side effects, tremors are the most abundant symptom, and after long-term therapeutical action, it is struma, as a consequence of hypothyreosis [111]. In men, lithium toxicity may lead to cardiotoxicity, development of fatal arrhythmias and sinus node disfunction. Renal toxicity increases water and Na diuresis, dehydration, and acidosis [5].

Significant intoxications appear at 11–14 mg/L in serum, causing vomiting, diarrhea, tremor, and tiredness and finally leading to reduced consciousness and hypertonic muscle reactions. In the case of less than 14 mg/L in serum, NaCl infusion can be applied as a therapeutical measure. In the case of higher levels, hemodialysis is necessary and may be repeated to cope with Li storage in various organs, e.g., in the brain and central nerval system [111].

In a group of former drug users receiving 400 µg Li per day in yeast, good mood symptoms like happiness, friendliness, and energy increased, whereas a control group showed no changes [7].

Li ions are rapidly absorbed from the gastrointestinal tract and peak in the blood plasma 2–4 h after administration. They do not bind to proteins in the gastrointestinal tract. Mild toxicity appears at up to 17 mg/L in serum, and acute intoxications have been reported at 42–67 mg/L. Lithium is transported across cell membranes by Na^+^-Li^+^ counter transport, which moves Na towards the intracellular compartment [105].

Ingested Li as its soluble salts is quantitatively absorbed from the small intestine via the Na^+^-channels and primarily by the kidneys. Highest accumulations were found in the cerebellum, followed by the cerebrum and the kidneys, in women 10–20% more than in men, but 13% less in the pancreas. Levels in the liver, lungs, ribs, and thyroid were about equal. At the end of the first trimester of embryonic development, organ lithium levels reach maxima, and they decline during the first 5–10 years of life. At equal daily Li dosages, body weight and height of the subjects were inversely correlated with serum Li [7].

## 7. Human Nutrition

An adult is estimated to contain 0.8 µg Li in 2.5 L of serum and 2.8 mg Li in the whole body, with a daily intake of 100 µg Li [112].

In human nutrition, primary dietary sources are grains and vegetables but also tap water and beverages [7]. Lithium is probably essential, and the minimum requirement for adults has been estimated at <100 µg/d, but this is still highly controversial.

Table 10 gives an overview of data from various countries. Differences between locations seem larger than differences between food items. Special Li sources seem to be insects and fish, and minima occur in sugar, honey, and fat. No data about Li contents in commercially available table salt could be found, as this is not labeled among quality criteria. Li contents in animal feed salt from Austrian salt mines is rather low at 15 µg/kg (unpublished results). A provisional investigation of commercially available Austrian table salt resulted in finding amounts of 0.86 and 1.64 mg/kg, and commercially available sea salt contained 0.31 mg/kg and 0.43 mg/kg. Thus, the contribution to the average daily intake in Austria from 6–7 g table salt [113] is about 8 µg, which is within the amount from solid food. However, as intake and sources of salt may vary globally, this has to be investigated in a more profound way.

In the UK, Li concentrations in various food items were slightly above detection limit at this time, and the daily intake was estimated at 18 µg Li [115]. Similarly, a total diet study analyzing dietary exposure from prepared and cooked meals, sampled in 1994, estimated a mean daily intake of 17 µg Li in the UK for adults, as well as a maximum of 29 µg [116]. In addition, in a French total diet study treating 998 composite samples for 300 individual food items, Li was close to the detection limit, resulting in a median of 7 µg/kg fresh weight. From this, a daily intake of 18 µg (range 7.8–144) Li for adults and 11 µg (range 4.6–38 µg) for children has been achieved. The main sources were soups, coffee, and bread [117].

For Austria, using the data of Table 10 and the average annual intake, an average daily intake of about 10.8 µg Li (without eggs, fat, and drinks) can be estimated. The main sources of this are cereals and potatoes, but a consumption of 2.5 L of water or drinks might contribute more, in particular if mineral water is consumed.

The main intake seems to come from water and drinks. In Austria, the annual average consumption per capita is 98.4 L beer, 26.4 L wine, and 18.4 L fruit juices [137]. In Germany, respective data have been reported as 154 L mineral, spring, and table water, 164 L coffee, 123 L soft drinks, 102 L beer, 24 L wine, and 80 L milk. Whereas an ample range in water has been reported (median 31.4 µg/L), beer (8.2 µg/L) and wine (6.0 µg/L) were low, and coffee brewed from deionized water was negligible [138], which results in about 18 µg/day from the drinks, but this is largely dependent on consumption of mineral waters (see Section 5.2).

Annual consumption of bottled water in Serbia has been reported at 75 L [100].

### 7.1. Fruits and Vegetables

The mean lithium in cloudy varietal apple juices from Klosterneuburg (Austria; flysch area) was 0.9 µg/L (range 0.3–2.3) [139].

Lithium in apple fruits (variety Topaz) from different years but the same site remained within the same range in spite of different weather conditions, contrary to the leaves. Lithium in apple leaves had a slight tendency to be higher from seedling rootstocks than from crafted plots [140,141].

In edible parts of cabbage and sprouts grown in Denmark, frequency distributions of Li concentrations were asymmetric, and thus a median is given in Table 10 [126]. Li concentrations in onions from 11 farms in Funen and Jutland correlated positively with soil pH and slightly negatively with soil clay, but not with soil organic carbon [124].

In onions grown in Hokkaido (Japan), using ammonium sulfate as a fertilizer lowered the uptake of Li and Tl into onions but increased Rb [125].

Tomatoes (17 varieties, three substrates) contained median Li at 7.7 µg/kg (9.6 ± 8.2) wet weight resp. 92 µg/kg (25–310) dry weight. Li analyzed including the seeds tended to be less, like alkaline earths, whereas other trace elements tended to be more [123].

In order to test moringa seeds as a possible food supplement and source of essential trace elements in Southern Africa, seeds of *Moringa ovalifolia* from three locations in Namibia and seeds of Moringa oleifera from Limpopo in South Africa were analyzed for main and trace elements. Whereas most essential micro elements varied according to the site, concentrations of Li (10.8 mg/kg dry weight), Ba, Si, Al, and Zn were equal. Beneath high enrichment of Li, Cd, and Ni, others like Co, Mn, Zn, Cu, and Fe were found at levels typical for green plants [129].

In the herbal mixture “Species thymi composita,” quite high Li content of 5.86 mg/kg was found, but the aqueous extract was below the detection limit [128].

Though honey is of animal origin, some legislation treats it separately from meat. In honeydew, the level of all trace elements is usually higher than in floral honey, and just for B and Ca, it is the opposite. Against this trend, linden honey contained more Li, Na, and B than honeydew [130,142].

### 7.2. Meat and Fish

Lithium in meat and liver has been found at the same low level throughout [118,121,122]. Enhanced ingestion primarily targets the kidneys. More Li can be expected in fish and shellfish (see Table 10).

In the future, insect-based food may partially replace traditional animal protein sources in Europe, like it is already accepted in Southeast Asia, Mexico, and Africa. In multi-element analysis of 31 commercially available ready-made insect-containing items, Li contents were higher in products from Asia and Latin America than from Europe. Though bioaccumulation patterns vary by insect species and development stage, mean and range can be given as 0.10 ± 0.10 mg/kg dry weight for edible insects and 0.06 ± 0.05 mg/kg dry weight for insect-based food items [133].

### 7.3. Milk and Dairy Products

Lithium in raw milk sampled on farms in Lower Austria contained a median of 2.7 µg/L (range 0.4–8.4), respectively 20 µg/kg (range 3–61) in dry weight. Data from different areas and cow species were overlapping, but there was a slightly higher trend in the molasse zone presumably due to soil clay minerals [143].

Like for most element contents (except K and Zn), Li contents increase from cow to goat to sheep milk (yogurt) [119,144] until horse milk at 15 ± 1 µg/L wet weight [145]. In cow milk, median Li of 2.1 µg/kg (range 1.2–5.1) wet weight was found, in goat milk 5.5 µg/kg (range 3.9–7.9), and in sheep yogurt 8.5 µg (range 5.5–22.9). Alternatively, vegan soy drinks contained a median of 10.0 µg/L (range 1.9–18.8) [119].

Whereas the Austrian cow milk samples corresponded to 18 ± 9 µg/kg dry weight, commercial milk powder purchased in Tanzania was a bit higher in Li content, at a median of 32 µg/kg (range 4–148) dry weight [146]. In Hungary, Li amounts in cream and cheese were found slightly enriched versus raw milk, with respect to wet weight [120,147].

Concentrations of lithium in human milk from healthy mothers were about the same as in serum and whole blood [112]. Lithium in human breast milk from Japan was 7.9 ± 1.6 µg/kg (range 3.3–10.7 µg/kg). No trend with the days postpartum nor differences between summer and winter were observed [148].

Human milk from 30 mothers living in Turin and its surroundings during the second month of lactation, sampled during five subsequent days, contained Li at 1.0 ± 0.4 µg/L. This was similar to levels reported from other countries. As a result of size-exclusion chromatography, Li appeared to be equally distributed among components with high and low molecular weight [149].

In and around Graz/Austria, Li in human milk ranged within < 0.08–1.3 µg/L. There was no significant change from the colostrum to the last stage of maturity, which was also found in the cases of Be, Bi, Cd, Co, Hg, La, Mn, Sb, Tl, and Zn. The Li concentrations in serum (0.29–0.31 µg/kg) and whole blood (0.52–0.64 µg/kg) of the respective mothers were approximately in the same range. A healthy solely breastfed baby is taking in 150–180 mL milk per kg body weight during 24 h [112,150].

If lithium is applied as an antidepressant drug, the excretion of lithium into human breast milk is low, but lithium has a long elimination half-life in the infant, and thus the relative dose for the infant is high. Lithium-induced toxicity in the infant as a result of a high level in breast milk effects the kidneys and the thyroid gland and leads to hypotonics and cyanotoxicity [151]. The milk-to-plasma concentration ratio ranges between 0.2 and 0.7 [151].

Plasma lithium peaks are reached 2–4 h after administration. Mild toxicity appears at levels up to 18 mg/L in the serum [105]. When lithium was given in therapeutic doses to treat bipolar disorder of the mother within the range of 600–1500 mg per day, 12.2 ± 8.5% (range 0–30%) of the ingested dose was transferred to the breast milk, estimated via analysis of milk and infant serum [152].

### 7.4. Wine

Lithium, as well as B, Mg, Ca, Rb, Cs, and Pb seem to be constant throughout the winemaking process and to be independent of the addition of bentonites. This was concluded from sampling the winemaking process of five German white wines from five regions, starting with fermentation with yeast, addition of K_2_S_2_O_5_, fining with NaCa-bentonite, and clarification with Ca-bentonite. Li concentration in must was 16.4 µg/L [131].

Most of 24 white and 16 red wines from several countries ranged within 2.3–15 µg/L, and just Rioja from Spain was higher, within 43.6–48.1 µg/L, along with one from Rhine-Hesse at 40.2 µg/L [131].

Lithium was one of the variables utilizable to discriminate wines from four different regions of Bohemia by principal component analysis. White wine from the Mostecko region contained 240 µg/L, contrary to wine from Zernosecko (11 µg/L), Roudnicko (10 µg/L), and Melnicko (4.7 µg/L) on average. Similarly, red wine from the Mostecko region contained 160 µg/L, contrary to wine from Zernosecko (12 µg/L) and Roudnicko (14 µg/L) [132]. In addition, in northwest Spain, 22 red wines from the location Ribeiro Sacro contained less Li (19 ± 3 µg/L) than 17 red wines from other locations in Galicia (35 ± 11 µg/L) [136].

Among several elements obtained from multi-element analysis, red wines from the four wine-producing countries in South America could be discriminated by their contents in Li-Mg-Rb-Tl-U data, but not by the lanthanides. Li correlated negatively (r = −0.939) with Rb, but not significantly with other ions. Means of Li in red wines were 189 (range 71–354) from Argentina, 8 (range 2–27) from Brazil, 9 (range 3–14) from Chile, and 28 (range 18–42) from Uruguay, in µg/L [135].

### 7.5. Mushrooms

In the case of mushrooms, trace element contents in general presumably reflect the geochemical characteristic of the site. In 15 edible wild mushroom species sampled in forested areas of Zagreb city and Karlovac County, the group of lithogenic elements Al-Ba-Ti-Li-Rb-Cs was positively correlated with each other, indicating a common mechanism of their uptake. Li occurred in a rather narrow and non-accumulating concentration range of a median 29 µg/kg dry weight (range 10–79) [134].

Edible mushrooms from pasture lands, forests, and lawns in the western Black Sea region of Turkey contained 94–186 µg/kg Li in dry weight [127].

## 8. Conclusions

By 2030, 11 million tons of spent lithium batteries have been predicted globally due to the increasing use of electrically driven cars, apart from other technical uses. The respective batteries contain 5–7% Li, which is more than in the richest ores, and additionally in a quite mobile form. Before the onset of diffuse pollution due to illegal disposal and lack of recycling, a compilation of current baseline data seems necessary. As an analyst, this is an occasion to present available data together with personally obtained data, particularly those that are not accessible via the abstracts. Because Li does not range within main elements, nor heavy metals, nor nutrients, it has not been requested among quality criteria, and it has rarely been ordered to be analyzed or labeled.

Mobilization of Li from rocks and storage in green plants and animal tissues is low, but solubility is high. Thus, the potential of dissipation and passage of waste treatment plants is high, but in this field, gaps of knowledge still exist.

Whereas domestic animals (at least in central Europe) receive feeds and salt at low Li levels, in the case of human nutrition, it turns out that the main sources are table salt and mineral water, of which source and uptake may vary widely. To the contrary, the contribution of solid nutrition is on the average about 10–11 µg per day. This makes the estimation of the average daily intake highly uncertain and necessitates double portion studies. In addition, former studies correlating Li in tap water with suicide rates seem doubtful, because many people drink bottled water, beer, and juices instead. Additionally, differences in dietary intake between wet and dry haline regions need to be investigated in future works.

Therapeutic use of LiCl has been known for a long time, and debates about essentiality and toxicity seem controversial to a non-toxicologist. Some aspects have been outlined and references given. However, the discussion would be more profound if the individual baseline intake was better known.

## Figures and Tables

**Table 1:** Li in marine surface sediments close to northern Japan [33].

mg/kg	Min	25%	Median	Mean	75%	Max
Hokkaido—coastal	5.2	19.4	28.0	26.8	32.9	68.0
Hokkaido—terrestrial	5.0	16.8	25.6	25.9	34.3	56.9

**Table 2:** Surface sediments of the Pacific, Li mg/kg [34].

Zone	<1 µm	1–10 µm	Japanese Side	Mexican Side
Volcanic-terrigenic and biogenic precipitates of the shelf	37	30	20	54
Hemipelagic clayey weakly siliceous layers	55	30	33	54
Pelagic clays of transient type	71	46	40	57
Pelagic red clays of deep sea with volcanic ashes	77	46	56	50
Pelagic red clays of deep sea with zeolites	70	59	57	61
Gravel and pebbles of submarine underwater hills	-	-	41	-
Precipitates of the Hawaiian archipelago containing volcanoclasts	-	-	31	31

**Table 3:** Means of total Li concentrations mg/kg in Danube sediments [39].

mg/kg	Aschach	Ottensheim	Abwinden	Wallsee	Ybbs	Melk	Altenwörth	Greifenstein
63–250 µm	28	29	33	30	29	31	31	33
20–63 µm	22	21	24	24	24	24	25	25
<20 µm	45	43	45	47	46	43	43	42

**Table 4:** Li occurrence and nutrient-related input from fertilizers, after [54].

	mg/kg	Range	g for 100 kg N	Range	g for 100 kg P	Range	Samples
Ammonium nitrate lime	0.34	0.12–0.65	0.13	0.03–0.24			22
K-salts	0.42	0.09–5.31					18
NPK + sulfate	0.91	0.14–3.38	0.67	0.11–2.60	2.06	0.53–12.3	398
PK fertilizer	0.96	0.40–3.05			1.66	0.79–3.85	110
NPK fertilizer	0.99	0.04–4.13	0.65	0.02–3.09	2.09	0.06–12.6	362
Super phosphate	1.28	0.94–3.51	0.54	0.43–0.76	1.84	1.18–2.69	12
Lime, dolomite	1.99	0.48–21.1					77
Rock phosphate	2.32	1.55–3.60			1.90	1.32–3.59	
Di-ammonium phosphate	3.00	1.29–4.55	1.65	0.62–2.53	1.47	0.61–2.51	88
Triple phosphate	3.00	1.85–7.48			1.32	0.89–4.76	81
Manures and dungs	3.71	0.18–10.9	3.69	0.38–44	26.9	2.89–115	
Garden molds	5.36	0.22–12.0			341	33.8–1293	
Composts	14.1	10.2–25.8	70	24–350	353	65–1129	

**Table 5:** Li mg/kg in soils.

	Soil Type	Median//Range	Digestion	Reference
New Zealand	Silty clay loam	48.8 ± 11.1	HCl-HNO_3_	[20]
New Zealand	Clay loam	24.9 ± 4.3	HCl-HNO_3_	[20]
New Zealand	Silt loam	24.3 ± 4.4	HCl-HNO_3_	[20]
New Zealand	Loam	21.6 ± 2.7	HCl-HNO_3_	[20]
New Zealand	Sandy loam	11.7 ± 2.6	HCl-HNO_3_	[20]
New Zealand	Sandy clay loam	10.9 ± 3.2	HCl-HNO_3_	[20]
Ottawa Canada	Urban soil	10.5//7.4–18.5	HF-HNO_3_-HClO_4_	[62]
Xuzhou China	Urban soil	36//25–47	HF-HNO_3_-HClO_4_	[63]
Poland	Sandy soil	8.55//4.0–26.0	HF-HCl-HNO_3_-HClO_4_	[57]
Poland	Silty and loamy soils	21.0//10.0–45.0	HF-HCl-HNO_3_-HClO_4_	[57]
Japan	Andosols	22 ± 11//1.6–58	HF-HNO_3_-HClO_4_	[58]
Japan	Cambisols	39 ± 23//2.2–110	HF-HNO_3_-HClO_4_	[58]
Japan	Gleysols	43 ± 18//15–98	HF-HNO_3_-HClO_4_	[58]
Japan	Acrisols	42 ± 21//5–110	HF-HNO_3_-HClO_4_	[58]
Austria	Apple orchard soils	35//29.6–52.6	HF-HNO_3_-HClO_4_	[61,64]
Austria	Apple orchard soils	21.1//15.9–33.7	KClO_3_-HNO_3_	[61,64]
Carinthia Austria	Forest soils	17.2//9.6–30.3	HCl-HNO_3_	[60]

**Table 6:** Lithium in composite feeds (mg/kg in dry mass) [73].

Composite Feed	Median	Range	Number of Samples
For calves	0.945	0.150–1.46	12
For sheep and goats	1.32	0.160–2.93	15
For pigs and sows	0.66	0.254–1.77	27
For piglets	0.935	0.161–2.50	60
For poultry	0.945	0.257–3.09	46
For cats and dogs	0.800	0.126–5.65	35
Hay + grass silage	0.44	0.031–3.56	81
Maize silage	0.100	0.023–0.24	13
Apple leaves	0.136	0.045–0.628	133

**Table 7:** Lithium in supplementary feeds (mg/kg in dry mass) [73].

Supplementary Feed	Median	Range	Number of Samples
For cattle	0.94	0.041–3.41	85
For calves	1.46	0.410–5.84	39
For horses	1.05	0.363–4.29	45
For pigs and sows	1.57	0.387–4.96	166
For piglets	1.70	0.232–4.64	122
For poultry	1.93	0.362–9.90	42

**Table 8:** Li in surface waters, µg/L.

Location	Samples	Median	Range		Reference
Freshwater reference		3			[77]
Denmark	139	10.3	<5–30.7		[79]
Latvia	720	1.3 shallow wells 1.9 springs 5.5 confined aquifers		<0.45 µm	[80]
Greece	149	11.1	0.1–121		[81]
Austria	6460	11.3	<1–1300		[82]
Eastern England	47		<1–21		[83]
Romania		1.7	Qu: 0.71–5.8	springwater	[18]
Jizera peatland CZ		0.35 ± 0.21 precipitation 1.87 ± 0.23 shallow groundwater 3.10 ± 0.36 outflow			[84]
Massif Central F			0.07–195		[85]
Texas	3123		2.8–219		[86]
Danube		7			[87]
Oceans			170–180		[5]
Puna highland (NW Argentina)		81	8–1003	Urban + 5 villages	[88]

**Table 10:** Li in human nutrition.

Bakery Products	µg/kg		Reference	Country
Wheat	10.5//1–92	Dry weight	[114]	A
Cereals	34.6	Wet weight	[115]	UK
Bread	10	Prepared meal	[116]	UK
Cereals	20	Prepared meal	[116]	UK
Bread	27	Prepared meal	[117]	F
Breakfast cereals	9	Prepared meal	[117]	F
Pasta	2	Prepared meal	[117]	F
Rice and semolina	4	Prepared meal	[117]	F
Biscuits	5	Prepared meal	[117]	F
Cakes	12	Prepared meal	[117]	F
**Milk and dairy**				
Milk	3	Prepared meal	[116]	UK
milk	2.1	Wet weight	[115]	UK
Milk	6	Prepared meal	[117]	F
Raw Milk	19.5//27–63	Dry weight	[118]	A
Raw milk	25.5 ± 0.4	Wet weight	[119]	H
Skimmed milk	26.3 ± 0.2	Wet weight	[119]	H
cream	34.2 ± 0.3	Wet weight	[119]	H
Dairy products	5	Prepared meal	[116]	UK
Fresh dairy products	4	Prepared meal	[117]	F
Cheese (trappista, hajdu)	96–98	Wet weight	[119]	H
Cheese	10	Prepared meal	[117]	F
Hard cheese	13.8	Wet weight	[120]	A
Semi hard cheese	11.7	Wet weight	[120]	A
Soft cheese	21.1	Wet weight	[120]	A
Curdled milk cheese	6.9	Wet weight	[120]	A
Processed cheese	41.6	Wet weight	[120]	A
Cream cheese cow	12.1	Wet weight	[120]	A
Sheep + goat cheese	7.8	Wet weight	[120]	A
**Meat**				
Fish	21.8	Wet weight	[115]	UK
Fish	60	Prepared meal	[116]	UK
Fish	30	Prepared meal	[117]	F
Shellfish	123	Prepared meal	[117]	F
Chicken breast	7.5//<1–19	Dry weight	[121]	A
Chicken drumstick	9.0//1.5–20	Dry weight	[121]	A
Poultry	10	Prepared meal	[116]	UK
Poultry and game	6	Prepared meal	[117]	F
Meat	2	Prepared meal	[117]	F
Meat products	10	Prepared meal	[116]	UK
Offals	41	Prepared meal	[117]	F
Pork	1.2//<1–1.4	Wet weight	[118]	A
Wild pork	3.5//3–4	Wet weight	[118]	A
Veal	1.3//<1–2.5	Wet weight	[118]	A
Beef	2.5//<1–3.7	Wet weight	[118]	A
Horsemeat	3.6//1.5–22.5	Wet weight	[118]	A
Deer	5.6//<1–22.5	Wet weight	[118]	A
Liver	2.9//1.4–16.6	Wet weight	[118]	A
Kidney	9.9//2.6–75.8	Wet weight	[118]	A
Sausage	14.8/4.7–34.5	Wet weight	[122]	A
**Fruits and vegetables**				
Fresh fruit	5	Prepared meal	[116]	UK
Fruits	13.9	Wet weight	[115]	UK
Fruit products	10	Prepared meal	[116]	UK
Fruits	7	Prepared meal	[117]	F
Apples	6.6//<1–24	Dry weight	[64]	A
Tomatoes	92//25–310	Dry weight	[123]	A
Potatoes	17.5//<1–338	Dry weight	[114]	A
Potatoes	10	Prepared meal	[116]	UK
Potatoes	16	Prepared meal	[117]	F
Root vegetables	7.4	Wet weight	[115]	UK
Carrots	71.5//48–118	Dry weight	unpublished	A
Radishes	361//137–1880	Dry weight	unpublished	A
Onions	0.64//<−5.2	Wet weight	[124]	DK
onions	3–9	Wet weight	[125]	J
sprouts	0.48//<−12.8	Wet weight	[112]	DK
Green vegetables	10	Prepared meal	[115]	UK
Cabbage	1.48//<−11.0	Wet weight	[126]	DK
Vegetable	14	Prepared meal	[117]	F
Other vegetables	11.3	Wet weight	[115]	UK
Other vegetables	30	Prepared meal	[116]	UK
Mushrooms	94–186	Dry weight	[127]	TR
Thyme	5860	Dry weight	[128]	H
Moringa seeds	10,800	Dry weight	[129]	Namibia
**Fat**				
Oils and fat	3	Prepared meal	[116]	UK
Oils, margarine	2	Prepared meal	[117]	F
Butter	2	Prepared meal	[117]	F
**Others**				
Feed salt (Bergkern)	15	Dry weight	unpublished	A
Table salt	860/1640	Dry weight	unpublished	A
Sea salt	310/430	Dry weight	unpublished	global
Eggs	70	Prepared meal	[116]	UK
Nuts	10	Prepared meal	[116]	UK
Nuts and oilseeds	22	Prepared meal	[117]	F
Chocolate	21	Prepared meal	[117]	F
Sugar	6	Prepared meal	[116]	UK
Sugars	2	Prepared meal	[117]	F
Honey–rapeseed	1.2//<1–4	Dry weight	[130]	A
Honey–honeydew	6.3//3.8–12	Dry weight	[130]	A
Beverages	3.7	Wet weight	[115]	UK
Beverages	4	Prepared meal	[116]	UK
Non alcoholic bev.	4	Prepared meal	[117]	F
Alcoholic bev.	3	Prepared meal	[117]	F
Beer (median)	8.3/1.9–19.9	Prepared meal	[131]	D
Coffee	6	Prepared meal	[106]	F
Coffee (from deionized water)	0.1	Prepared meal	[132]	D
Tea (from deionized water)	1.4	Prepared meal	[132]	D
Soups	38	Prepared meal	[117]	F
Edible insects	96 ± 96	Dry weight	[133]	global
Insect based food	57 ± 50	Dry weight	[133]	global
**Wine**				
Red wine	189//71–354		[134]	Argentina
Red wine	9//3–14		[134]	Brazil
Red wine	28//18–42		[134]	Uruguay
White wine	16.4		[131]	Germany
Red wine	35 ± 11	Galicia	[135]	Spain
Red wine	19 ± 3	Ribeiro Saco	[135]	Spain
Wine	6.0/2.0–48.1	40 samples	[132]	global
Wine	5.1/2.4–15.2 (40.2)	15 samples	[132]	D
White wine	240	Mostecko	[136]	CZ
	11	Zernosecko	[136]	CZ
	10	Roudnicko	[136]	CZ
	4.7	Melnicko	[136]	CZ
Red wine	160	Mostecko	[136]	CZ
	12	Zernosecko	[136]	CZ
	14	Roudnicko	[136]	CZ

## Data Availability

No new data were created or analyzed in this study. Data sharing is not applicable to this article.
